# The impact of public health interventions in the Nordic countries during the first year of SARS-CoV-2 transmission and evolution

**DOI:** 10.2807/1560-7917.ES.2021.26.44.2001996

**Published:** 2021-11-04

**Authors:** Sebastian Duchene, Leo Featherstone, Birgitte Freiesleben de Blasio, Edward C Holmes, Jon Bohlin, John H-O Pettersson

**Affiliations:** 1 Department of Microbiology and Immunology, The University of Melbourne at The Peter Doherty Institute for Infection and Immunity, Melbourne, Australia; 2 Department of Methods Development and Analytics, Division of Infectious Disease Control and Environmental Health, Norwegian Institute of Public Health, Oslo, Norway; 3 Department of Biostatistics, Oslo Centre for Biostatistics and Epidemiology, Institute of Basic Medical Sciences, University of Oslo, Oslo, Norway; 4 Sydney Institute for Infectious Diseases, School of Life and Environmental Sciences and School of Medical Sciences, University of Sydney, Sydney, Australia; 5 Zoonosis Science Center, Department of Medical Biochemistry and Microbiology, University of Uppsala, Uppsala, Sweden

**Keywords:** SARS-CoV-2, Nordic, transmission chain modelling, virus exportation

## Abstract

**Background:**

Many countries have attempted to mitigate and control COVID-19 through non-pharmaceutical interventions, particularly with the aim of reducing population movement and contact. However, it remains unclear how the different control strategies impacted the local phylodynamics of the causative SARS-CoV-2 virus.

**Aim:**

We aimed to assess the duration of chains of virus transmission within individual countries and the extent to which countries exported viruses to their geographical neighbours.

**Methods:**

We analysed complete SARS-CoV-2 genomes to infer the relative frequencies of virus importation and exportation, as well as virus transmission dynamics, in countries of northern Europe. We examined virus evolution and phylodynamics in Denmark, Finland, Iceland, Norway and Sweden during the first year of the COVID-19 pandemic.

**Results:**

The Nordic countries differed markedly in the invasiveness of control strategies, which we found reflected in transmission chain dynamics. For example, Sweden, which compared with the other Nordic countries relied more on recommendation-based rather than legislation-based mitigation interventions, had transmission chains that were more numerous and tended to have more cases. This trend increased over the first 8 months of 2020. Together with Denmark, Sweden was a net exporter of SARS-CoV-2. Norway and Finland implemented legislation-based interventions; their transmission chain dynamics were in stark contrast to their neighbouring country Sweden.

**Conclusion:**

Sweden constituted an epidemiological and evolutionary refugium that enabled the virus to maintain active transmission and spread to other geographical locations. Our analysis reveals the utility of genomic surveillance where monitoring of active transmission chains is a key metric.

## Introduction

Following its initial description in December 2019, severe acute respiratory syndrome coronavirus 2 (SARS-CoV-2), the causative agent of coronavirus disease (COVID-19) [1,2], rapidly led to a major global public health event. Until 18 October 2021, the pandemic has caused more than 240 million infections and over 4.9 million deaths worldwide and had a significant impact on healthcare systems, societies and the global economy. Countries were struggling with how to effectively counteract the pandemic, balancing the protection of health with social and economic considerations. During the first year of the pandemic, in the absence of specific therapies and vaccines, efforts centred on non-pharmaceutical interventions, including initial short-term and large-scale restrictions to population movement (e.g. lockdowns), increased testing and various levels of physical distancing. Analysing local epidemiological consequences of control strategies, via their reflection in the short-term evolutionary dynamics of the virus, provides key information on the most effective approaches to reduce rates of transmission during emerging viral epidemics.

The Nordic countries, defined here as Denmark, Finland, Norway, Iceland and Sweden, provide an example of geographically, politically and socially related countries that employed different COVID-19 control strategies. Responses differed markedly during the first 6 months of the pandemic before becoming more similar in the following 6 months, which offered a natural comparison within and between Nordic countries. Sweden initially took a less restrictive approach based on recommendations, compared with legislative approaches in the other Nordic countries. In Sweden, recommendation-based measures did not enforce general population movement restrictions, schools for children younger than 16 years remained open, mandatory quarantine was not imposed for infected households and businesses continued to operate with adaptation to distancing limitations [3]. In contrast, Norway, Finland and Denmark imposed more invasive population movement restrictions that included enforced home office for workers in the public sector, schooling at home, targeted closure of businesses in the private sector, closing of restaurants, museums, sports centres, etc., as well as closed international borders for non-residents. Iceland, a small homogenous island population (of ca 350,000) never initiated population movement restrictions such as Norway, Denmark and Finland, but rather focused on large-scale testing and contact tracing to limit virus spread within the community. In relation to population size, Sweden has had a higher number of COVID19-related cases and deaths than the other Nordic countries [4,5], with a cumulative incidence of around 9,758 cases and 137 deaths per 100,000 people, compared with around 4,465 cases and 43 deaths in Denmark, 2,169 cases and 14 deaths in Norway, 1,608 cases and 17 deaths in Finland, and 1,790 cases and eight deaths in Iceland, by 2 May 2021 [6]; most cases occurred between April and July 2020 and between November 2020 to March 2021.

Although the relative success of COVID-19 control measures is normally gauged by the number of cases and deaths at the country level, interventions and mitigation strategies may lead to marked differences in transmission dynamics among populations, which are in turn reflected in the phylogenetic relationships between virus isolates. Using a comparative analysis of genome sequence data we addressed whether the different approaches to COVID-19 control employed by the Nordic countries resulted in differences in virus transmission dynamics. SARS-CoV-2 genome sequence data also allowed us to explore the relative frequencies of virus importation/exportation (i.e. virus phylodynamics) during the first year (January 2020 to March 2021) of the pandemic. Accordingly, we compiled an extensive dataset of SARS-CoV-2 virus genomes and performed a phylo-epidemiological study to identify any differences in transmission chain dynamics between these countries.

## Methods

### Dataset construction

We downloaded all SARS-CoV-2 genomes that were complete and had high sequencing coverage available on 22 March 2021 at the GISAID platform (www.gisaid.org). We selected all sequences from the five Nordic countries: 50,126 from Denmark, 2,374 from Finland, 4,167 from Iceland, 3,388 from Norway, and 7,863 from Sweden). To obtain a representative subset of the global diversity, we also selected 3,437 genomes from the latest NextStrain global build on 22 March 2021 [7].

Sequencing intensity was markedly different between countries, with Denmark and Iceland sequencing around 22% and 68%, respectively of reported cases. In Norway, Finland and Sweden, the sequencing intensity was 4%, 3% and 1.1%, respectively, during our period of sampling (Table). We attempted to control for such sampling bias by subsampling genomes relative to the Nordic country with the lowest sampling intensity. This means that all countries were sampled at a rate relative to Sweden of 1.1 genomes per 100 cases. For this purpose, we divided the number of genomes from each country by the cumulative number of cases reported in each country by mid-March 2021, as recorded in ourworldindata.org [8]. We repeated this procedure 10 times, and in each obtained a sequence alignment with all data from NextStrain and with the number of genomes from the Nordic countries proportional to the prevalence of the virus (Table). The complete dataset consisted of 71,355 genomes, 67,918 of them from the Nordic countries. Our 10 alignments with equal sequencing intensity for the Nordic countries had between 15,616 and 15,297 sequences (i.e. between 12,179 and 11,860 Nordic genomes, plus the 3,437 from the NextStrain build). The GISAID accession numbers and details of all sequences included in the final sequence alignment are available in Supplementary Table S1.

**Table:** Summary statistics computed for SARS-CoV-2 genomic data, Nordic countries, 22 March 2021 (n = 67,918 genomes)

	Denmark	Finland	Iceland	Norway	Sweden
TMRCA range of first detected transmission chain	14 Jan–5 Mar 2020	24 Jan–4 Mar 2020	20 Feb–13 Jul 2020	29 Jan–20 May 2020	1 Jan–29 Jan 2020
Median TMRCA of transmission chains (range)	22 Oct 2020 (20–25 Oct 2020)	26 Jul 2020 (17 Jul–14 Aug 2020)	19 Jun 2020 (6 Jun–25 Sep 2020)	13 Nov 2020 (28 Oct–18 Nov 2020)	13 Oct 2020 (10–15 Oct 2020)
Median number of genomes included after correcting for prevalence (range)	2,380 (2,341–2,457)	773 (746–799)	70 (56–72)	940 (854–988)	7,863 (7,863–7,863)
Median number of transmission chains (range)	227 (197–231)	86 (79–103)	4 (3–5)	105 (94–119)	677 (638–690)
Median duration of transmission chains in days (range)	42 (40–44)	26 (21–28)	45 (19–56)	25 (23–27)	31 (30–32)
Median size of largest transmission chain (range)	222 (99–243)	80 (65–135)	44 (34–56)	134 (129–147)	366 (175–368)
H_t_-index (range)	21 (20–22)	11 (9–12)	2 (2–3)	11 (10–12)	38 (36–39)
Median number of exportation events (range)	125 (98–203)	36 (20–60)	0.002 (0.00–0.80)	33 (22–42)	552 (479–704)
Median number of importation events (range)	385 (361–412)	248 (230–271)	18 (14–24)	264 (246–273)	579 (491–628)
Ratio exportations : importations (range)	0.34 (0.24–0.56)	0.14 (0.0813–0.26)	0.00 (0.00–0.057)	0.13 (0.083–0.17)	0.95 (0.76–1.45)
Number of genomes collected	50,126	2,374	4,167	3,388	7,863
Genome collection date range	2 Mar 2020–21 Feb 2021	29 Jan 2020–6 Feb 2021	2 Mar 2020–5 Jan 2021	29 Jan 2020–8 Mar 2021	31 Jan 2020–9 Mar 2021
Proportion of genomes per confirmed case	0.22	0.03	0.68	0.04	0.01
Number of cases reported by 22 Mar 2021 sampling collection date	226,777	72,073	6,119	87,519	744,272
Cumulative number of cases per million people by 22 Mar 2021^a^	39,129	12,990	17,821	16,107	73,254
Transmission chain ca detection date^b^	4, 0	8, 1	NA, NA	6, 1	2, 0
Transmission chain duration ca detection date^b^	3, 0	8, 0	NA, NA	3, 1	8, 1

H_t_: number of transmission chains with at least H_t_ cases; NA: not available; TMRCA: time to the most recent common ancestor.

^a^ Source: [8].

^b^ Number of replicates out of 10 with positive slope; before and after early November 2020.

The median and range (over 10 replicates) is shown for relevant statistics, with the exception of the TMRCA (the putative time of emergence) of the first transmission chain, for which only the range is reported because the average disproportionally affects countries with low prevalence. For regression statistics, the symbol ca denotes ‘function of’, and in these cases we report the number of replicates with positive regression slopes before and after 31 August 2020.

### Estimating a time-scaled phylogenetic tree

We estimated maximum likelihood phylogenetic trees using IQ-TREE v2.0.6 [9] employing the GTR + Γ model of nucleotide substitution [10]. We fit a strict molecular clock to the data using LSDv0.3 [11]. We calibrated the molecular clock by specifying the sequence sampling times and fixing the evolutionary rate to 1 × 10^− 3^ nucleotide substitutions per site per year as estimated previously [12]. We chose this approach because testing for temporal signal and fitting a sophisticated molecular clock model is not feasible for a dataset of this size. For a small number of sequences (n = 113; 0.16% of the complete dataset) only the month and year were available, therefore we assigned them the 15th of the corresponding month.

### Detection of virus transmission chains

We defined transmission chains as monophyletic groups of genomes (at least two) collected from one of the Nordic countries, which is analogous to transmission lineages as defined previously [13]. We computed key statistics from each transmission chain, specifically: the duration (i.e. the length of time in days from the first to the last collected genome), the size (i.e. the number of genomes) and the time of origin (i.e. the time to the most recent common ancestor (TMRCA)). For each country, we also calculated an H_t_-index of transmission chains, where H_t_ is the largest number of transmission chains with at least H_t_ cases. Larger values reflect an increase in the number and size of lineages. Because we subsampled the data 10 times (Supplementary Figure S1), we computed the range of values for these statistics, as a crude measure of uncertainty due to sampling.

We fit linear regressions for transmission chain size against the detection date (the date of collection of the first genome within a transmission chain), such that positive trends would suggest increased community spread over time (i.e. larger transmission chains over time). We also regressed the duration of transmission chains against detection date, where a positive trend means that transmission chains detected later in the pandemic tended to last longer. Because the number of cases had substantially decreased around August 2020, we fit our regressions across two periods: before and after 31 August 2020. Importantly, the end of August also preceded by a few months the emergence of the Alpha variant of SARS-CoV-2 (Phylogenetic Assignment of Named Global Outbreak (Pango) lineage designation B.1.1.7) and its introduction to the Nordic countries (Supplementary Figure S6). This variant of concern has been shown to have increased transmissibility relative to circulating diversity [14]. We only report trends as residual distributions were strongly skewed.

### Inferring virus migration dynamics between Nordic countries

To infer the frequency of importation or exportation events of SARS-CoV-2 between the Nordic countries, as well as from the rest of the world, we employed a Bayesian stochastic mapping approach, also known as discrete phylogeography, as implemented in BEAST v1.10.4 [15-17] using guidelines from Dellicour et al. for very large genomic datasets [18]. We fixed the time-tree described above and assigned a geographical location for each tip, which could be either of the five Nordic countries or 'other' (for those collected in other countries). This method is broadly similar to that used to infer geographical movement of the virus in Belgium [12]. We repeated this procedure for the 10 subsampled replicate trees and report median posterior values and ranges across replicates. Stochastic mapping generates a posterior distribution of ‘type changes’ between locations along the branches.

We ran a Markov Monte Carlo chain with a length of 5 × 10^7^ steps, recording every 5,000th step. Sufficient sampling from the posterior was assessed by verifying that the effective sample size of all parameters was above 200 as estimated in Tracer v.1.7 [19]. We inferred the posterior number of migration events between the six possible states (Markov jumps) and the amount of time spent at each state, known as Markov rewards.

### Assessing public health measures and governmental stringency

To provide an overview of public health measures taken and how they were enforced between February 2020 and March 2021 per Nordic country, we plotted data from the Oxford COVID-19 Government Response Tracker [20] which included: testing policy, contact tracing, public information campaigns, international travel control, workplace closure, school closure, cancelation of public transportation, stay at home requirements, facemask usage, gathering restrictions, cancelation of public events, restrictions on movement and government stringency response index (Supplementary Figures S2–S5).

### Ethical statement

Ethical approval was not needed for this study. All the data are available from www.gisaid.org. 

## Results

**Figure 1:**
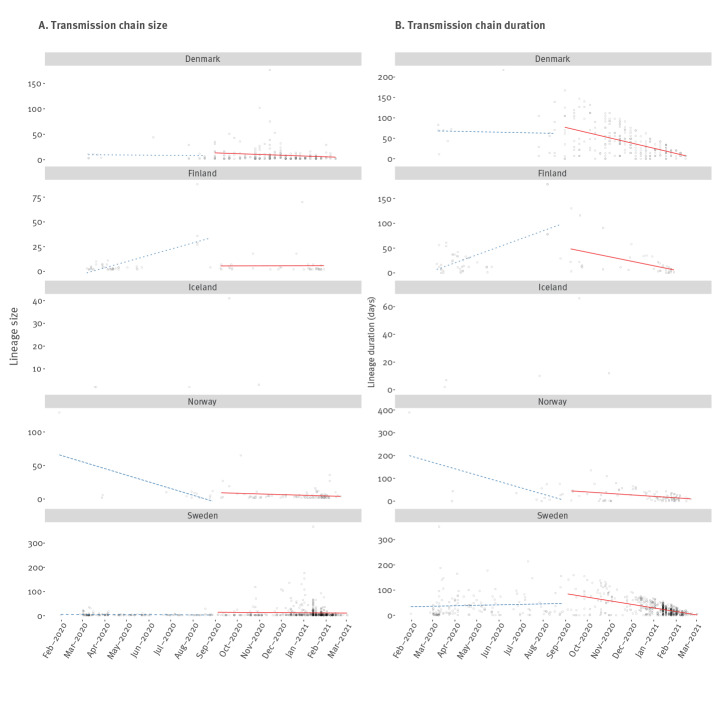
Summary of transmission chain size and duration against the time of the first sample from each chain, Nordic countries, up to 22 March 2021 (n = 67,918 genomes)

The estimated time of emergence of sampled transmission chains provides information about the onset of community transmission, although such an association is sensitive to sampling bias, particularly the timescale of country-specific sequencing efforts. Our phylogenetic analyses revealed that SARS-CoV-2 was imported to all Nordic countries between January and late February 2020, with detectable community transmission from early February in Sweden and from early March in the other Nordic countries as inferred by the presence of transmission chains (Figure 1). Sustained community transmission continued for all Nordic countries beyond April 2020, with the exception of Iceland, which was characterised by a relatively short 1-month period of community transmission, and Norway, which had a low number of cases combined with a low sequencing intensity of 0.04 (Table).

Trend lines are fitted here to one of 10 replicates, with dotted lines corresponding to trends that were not repeated in at least nine of 10 replicates. Trends are omitted for Iceland due to low sample size. Trends are split on either side of 31 August 2020.

**Figure 2:**
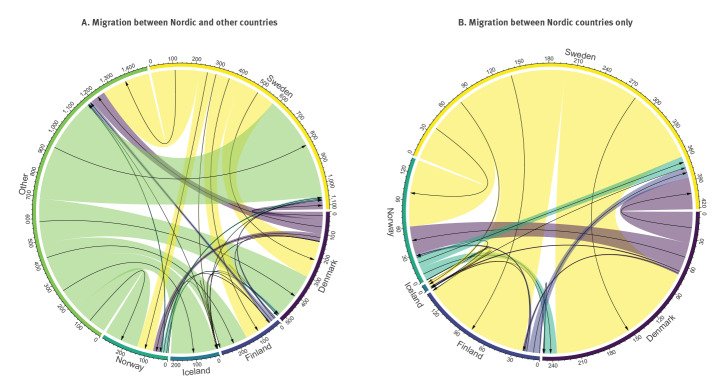
Number of migration events between the five Nordic countries and other locations, with sequences collected up to 22 March 2021 (n = 67,918 genomes)

The total number of exportation events was larger for Sweden (n = 552; range: 479–704) than for Denmark (n = 125; range: 98–203) (Table). Most exportation events from Sweden were into Finland, whereas those from Denmark were into non-Nordic countries, followed by Sweden and Iceland (Figure 2, panel B). An inspection of the amount of time in the tree occupied by each country, known as the Markov rewards, revealed that Sweden occupied the largest portion, consistent with it being the major exporter. In contrast, Norway, had very low Markov rewards, although it had more genomes than Iceland and Finland, both with higher Markov rewards (Supplementary Figure S8).

The size of the coloured links denotes the posterior median number of inferred migration events from the Bayesian phylogeographic analysis across 10 replicates. The black arrows represent the migration direction.

The number of transmission chains observed had a mean of 227 (range: 197–231) for Denmark, 105 (range: 94–119) for Norway, 86 (range: 79–103) for Finland and 45 (range: 19–56) for Iceland. The highest number of transmission chains – 677 (range: 638–690) – was observed in Sweden. The median duration of such transmission chains (number of days from detection to last sampled case) was higher in Iceland (n = 45; range: 19–56) and Denmark (n = 42, range: 40–44) than other localities: 31 (range: 30–32) for Sweden, 26 (range: 21–28) for Finland, and 25 (range: 23–27) for Norway (Table).

The largest transmission chain was found in Sweden, with 366 cases (range: 175–368), followed by Denmark with 228 (range: 99–243), Norway with 134 (range: 129–147), Finland with 80 (range: 65–135) and Iceland with 44 (range: 34–56). Sweden had the highest H_t_-index (Table). The H_t_-index was 38 for Sweden (range: 36–39), 21 for Denmark (range: 20–22), 11 for Finland (range: 9–12), 11 for Norway (range: 10–12) and 2 for Iceland (range: 2–3).

There was inconclusive evidence of an increase in transmission chain size in the first period (before 31 August 2020) when between two and eight of 10 subsampling replicates displayed a positive regression slope. Because sample size and number of data points in the second period were small, we did not perform a trend analysis for Iceland. After 31 August 2020, most replicates (nine or more) exhibited a negative trend for all countries, indicating a decrease in transmission chain size over time. Similarly, we did not find consistent trends for the duration of transmission chains in the first period whereas in the second period; they appeared to decline over time, with at most one replicate displaying a positive slope in Denmark, Finland, Norway and Sweden (Table, Figure 1). 

## Discussion

Until March 2021, Sweden experienced a greater number of COVID-19-related cases and deaths in relation to population size than the other Nordic countries [6]. Our genomic analysis shows that while SARS-CoV-2 was imported to all Nordic countries during the same time period in early 2020, Sweden experienced more transmission chains which also tended to be larger, suggesting that Sweden had the least-interrupted community transmission and more established community transmission during the sampling period relative to the other Nordic countries. In all Nordic countries, the size of transmission chains and their duration decreased in the second time period examined here.

Denmark and Sweden acted as exporters of SARS-CoV-2 to their Nordic neighbours. Conversely, Iceland, Norway and in particular Finland showed lower levels of virus exportation. At the same time, our analyses indicate that Finland was the main receiver of the exportations from Sweden, suggesting a near unidirectional mode of exportation between the two countries. It is important to note, however, that even with enforced recommendations, one might also have to consider aspects of urbanisation (differing population density not only at country level but also regionally within the countries) and geographical location that vary in these countries. Importantly, Denmark and Sweden are transit countries and the latter has borders with three of the other four Nordic countries.

During the first 6 months of the pandemic, Sweden did not enforce community mobility restrictions. Instead, mitigation efforts included, for example, recommendations to maintain physical distancing, work from home, limiting sizes of social events and moving to distance education for students above 16 years (Supplementary Table S2). Conversely, Denmark, Finland and Norway enforced population movement restrictions in their communities, closure of borders and government-run facilities [4]. These mitigation efforts were taken during both the first and second pandemic wave (Supplementary Table S2, Supplementary Figures S2–S5). During the second wave, which started in early September 2020 in the Nordic countries, Sweden additionally recommended limiting the number of people who meet within social circles and going into voluntary home-based quarantine if experiencing COVID-19-like symptoms. In early 2021, Sweden also recommended the use of protective face masks in public spaces, ca 5–6 months after a corresponding recommendation in the other Nordic countries. In Norway, locally-based contact tracing supported by a national contact tracing team, testing, isolation and quarantining has been the primary strategy since the summer of 2020. In contrast, the Danish strategy focused on extensive testing that was free-of-charge with readily accessible testing units (Supplementary Figure S2). Norway, Finland and Denmark enforced local contact-reducing measures during periods of outbreaks. Iceland, in a similar manner to South Korea, used large-scale testing and contact tracing combined with physical distancing and voluntary home-based quarantine without the need to regulate population movement (see also Supplementary Table S2) [21]. 

Overall, our results suggest that stringent public health interventions in the second period may have limited the spread of existing transmission chains. Thus, the increased number of cases in the second half of 2020 was most probably driven by virus importations that did not lead to transmission chains of increasing size and duration. This is consistent with the fact that the majority of importation events leading to transmission chains or singletons occurred in the second half of 2020 for most countries (Supplementary Figure S7). Thus, while regulating population movement appears to be an efficient method to reduce community transmission [22,23], other mitigation efforts are also viable options under certain circumstances. 

Norway experienced comparatively low numbers of transmission chains during the first 6 months of the pandemic. The bulk of detected transmission chains emerged in mid-April and towards the end of July 2020. However, this might in part reflect a delayed start of genome sequencing, which has increased substantially in all countries since that time, and such results should therefore be interpreted with caution. Indeed, we note that the sequencing intensity of cases in Norway was lower than in some of the other Nordic countries with similar case burdens. Thus, sequencing proportion bias per country needs to be taken into account when considering our results. To reduce effect of sequencing bias, we attempted to subsample our data to obtain even sequencing proportions. Nevertheless, data subsampling does not correct for other sources of bias, such as the decision to sample close contacts of a positive case for sequencing or not. When sample size per country increases with time, the effect of such potential sampling biases is expected to decrease.

Our results indicate that Sweden’s mitigation strategy had an impact on the epidemiological situation internally and across the Nordic region as a whole. Sweden received considerable attention for the number of deaths reported. The country had one of the longest durations of excess deaths and the highest case numbers between February and May 2020 [5] that particularly affected the population above 60 years of age [24]. The causes and mechanisms as to why Sweden experienced comparatively high periodic excess death will need to be studied carefully. Kamerlin et al. suggested that a greater level of self-isolation would probably have reduced the number of deaths in Sweden [25].

The effect of closing/opening schools is also likely to have contributed to the transmission dynamics [26,27]. In the case of the Nordic countries, it can be argued that by closing pre-, primary and secondary schools, as well as higher education for several weeks during the spring of 2020 (Supplementary Table S2, Supplementary Figure S3), Denmark, Finland and Norway also reduced contact and virus transmission in the adult population to a greater extent than Sweden. In Sweden, schools remained open for children younger than 16 years during the same period. However, although Sweden exhibited a greater number of cases than the other Nordic countries and our results are consistent with periodically higher community transmission in Sweden compared with the other Nordic Countries during the first 12 months of the COVID-19 pandemic, higher community transmission does not necessarily imply a causal relationship with excess deaths. While we employ a robust genomic-based modelling approach to study transmission chain changes, equating, or directly relating, such changes with the number of deaths may lead to incorrect conclusions as we have not taken into account any patient-related epidemiological parameters such as sex or age. Furthermore, it has also been argued that the larger number of deaths during the early phase of the pandemic in Sweden may have been influenced by how admission criteria to intensive care units were applied, particularly for the elderly population in care homes at that time [25]. Thus, official mitigation strategies and efforts alone are unlikely to explain epidemiological differences between countries, which were also impacted by practical enforcement and the compliance from the general public, businesses and healthcare providers. In addition, vaccinations began in late December 2020 for all Nordic countries, initially focusing on people older than 70 years and identified risk groups. At our last sampling date (22 March 2021), between 9.5% and 13% of the population had been partially vaccinated in these countries and less than 5.5% of the population in each country had been fully vaccinated [8]. It is therefore unlikely that the onset of vaccination had any significant impact on our analyses. 

A critical issue, albeit one that has received little attention, is that if transmission chains are allowed to remain active, they also provide increased opportunity for the virus to evolve and adapt to local populations, potentially acquiring mutations that in some way enhance fitness. Indeed, several variants of concern have independently emerged globally, with mutations that are associated with epidemiologically important properties. For example, the Alpha variant acquired mutations that significantly increased transmission potential [14] and the Beta variant (B.1.351) exhibited increased resistance against vaccines [28]. Clearly, mutations routinely appear in the SARS-CoV-2 genome [29], and it is important to continuously evaluate their functional relevance. 

In addition to providing increased evolutionary potential, sustained transmission chains also provide epidemiological refugia from which the virus can be transmitted to other localities. This is in line with our results showing that Sweden had a greater frequency of exportation events among neighbouring Nordic countries. As a consequence, interrupting and ultimately stopping transmission chains is not only important to minimise virus spread within populations, but also to reduce the chances for the virus to accumulate mutations. By 26 September 2021, between 61% and 80% of the Nordic population have been fully vaccinated against COVID-19 [8]. Vaccinations will help reduce transmission chains and protect vulnerable patient groups, although the emergence and spread of the Delta variant (B.1.617.2) has raised concern about long-term vaccine effectiveness. In addition, and independent of the variant, there is also concern about waning immunity [30]. Nevertheless, genome sequencing will continue to play an important role in monitoring potential vaccine escape mutants and SARS-CoV-2 genetic diversity.

## Conclusion

Our study highlights the utility of continuous genomic surveillance and retrospective studies to compare and understand differences in pandemic responses with respect to transmission dynamics. In particular, our data suggest that transmission chain monitoring may prove to be a useful metric in comparing the outcome of outbreak mitigation strategies.

## Supplementary Data

11177000 Supplementary_figures

## Supplementary Data

31399000 Supplementary_tables
