# Supplementary material for: The impact of public health interventions in the Nordic countries during the first year of SARS-CoV-2 transmission and evolution
Source: Euro Surveill. 2021 Nov 4;26(44):2001996. doi: 10.2807/1560-7917.ES.2021.26.44.2001996 (PMC8569925; doi:10.2807/1560-7917.ES.2021.26.44.2001996)

Supplementary Figure S1. Number of SARS-CoV-2 genomes sampled with time for all Nordic countries and global samples (i.e. 'other') included in the final alignment.

This supplementary material is hosted by Eurosurveillance as supporting information alongside the article "the impact of early public health interventions during the first year of SARS-CoV-2 transmission and evolution in northern Europe , on behalf of the authors, who remain responsible for the accuracy and appropriateness of the content.
The same standards for ethics, copyright, attributions and permissions as for the article apply. Supplements are not edited by Eurosurveillance and the journal is not responsible for the maintenance of any links or email addresses provided therein.

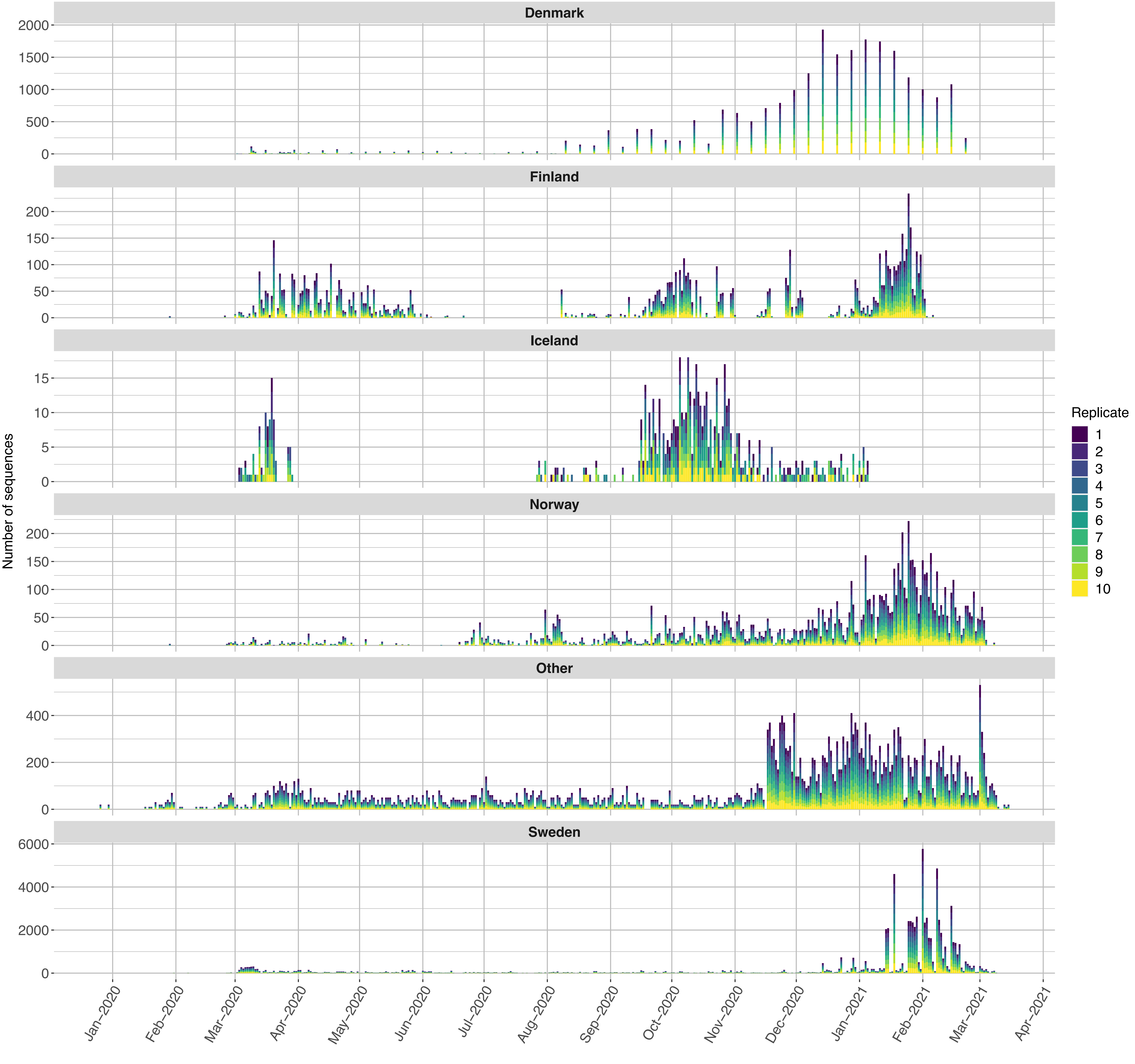

Supplementary Figure S2. Information on indicators of government response between February 2020 through March 2021 by country regarding (i) testing policy, (ii) contact tracing, (iii) public information campaigns, and (iv) international travel control.

This supplementary material is hosted by Eurosurveillance as supporting information alongside the article "the impact of early public health interventions during the first year of SARS-CoV-2 transmission and evolution in northern Europe", on behalf of the authors, who remain responsible for the accuracy and appropriateness of the content. The same standards for ethics, copyright, attributions and permissions as for the article apply. Supplements are not edited by Eurosurveillance and the journal is not responsible for the maintenance of any links or email addresses provided therein.

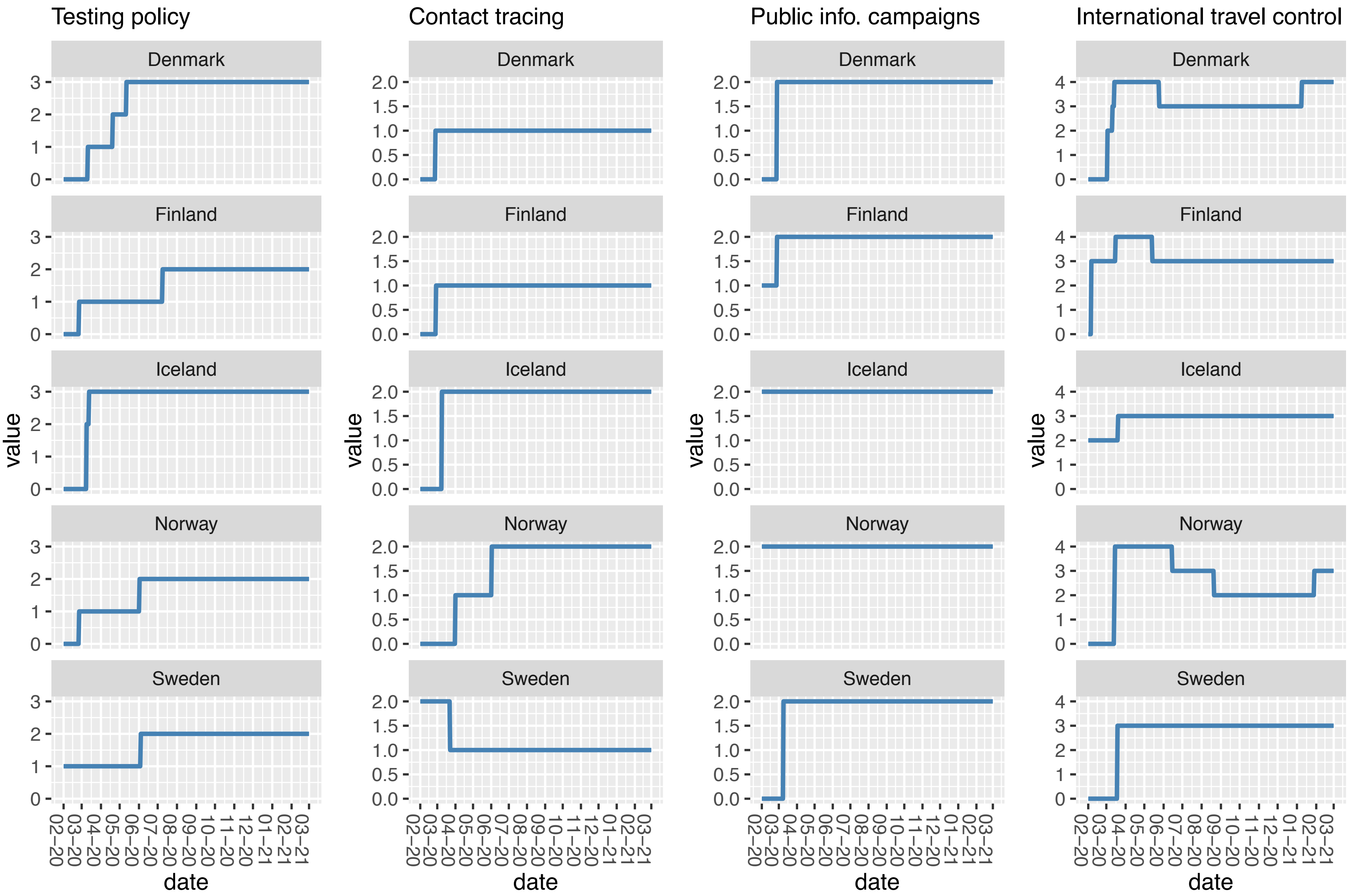

Supplementary Figure S3. Information on indicators of government response between February 2020 through March 2021 by country regarding (i) workplace closure, (ii) school closure, (iii) cancelation of public transportation, and (iv) stay at home requirements.

This supplementary material is hosted by Eurosurveillance as supporting information alongside the article "the impact of early public health interventions during the first year of SARS-CoV-2 transmission and evolution in northern Europe", on behalf of the authors, who remain responsible for the accuracy and appropriateness of the content. The same standards for ethics, copyright, attributions and permissions as for the article apply. Supplements are not edited by Eurosurveillance and the journal is not responsible for the maintenance of any links or email addresses provided therein.

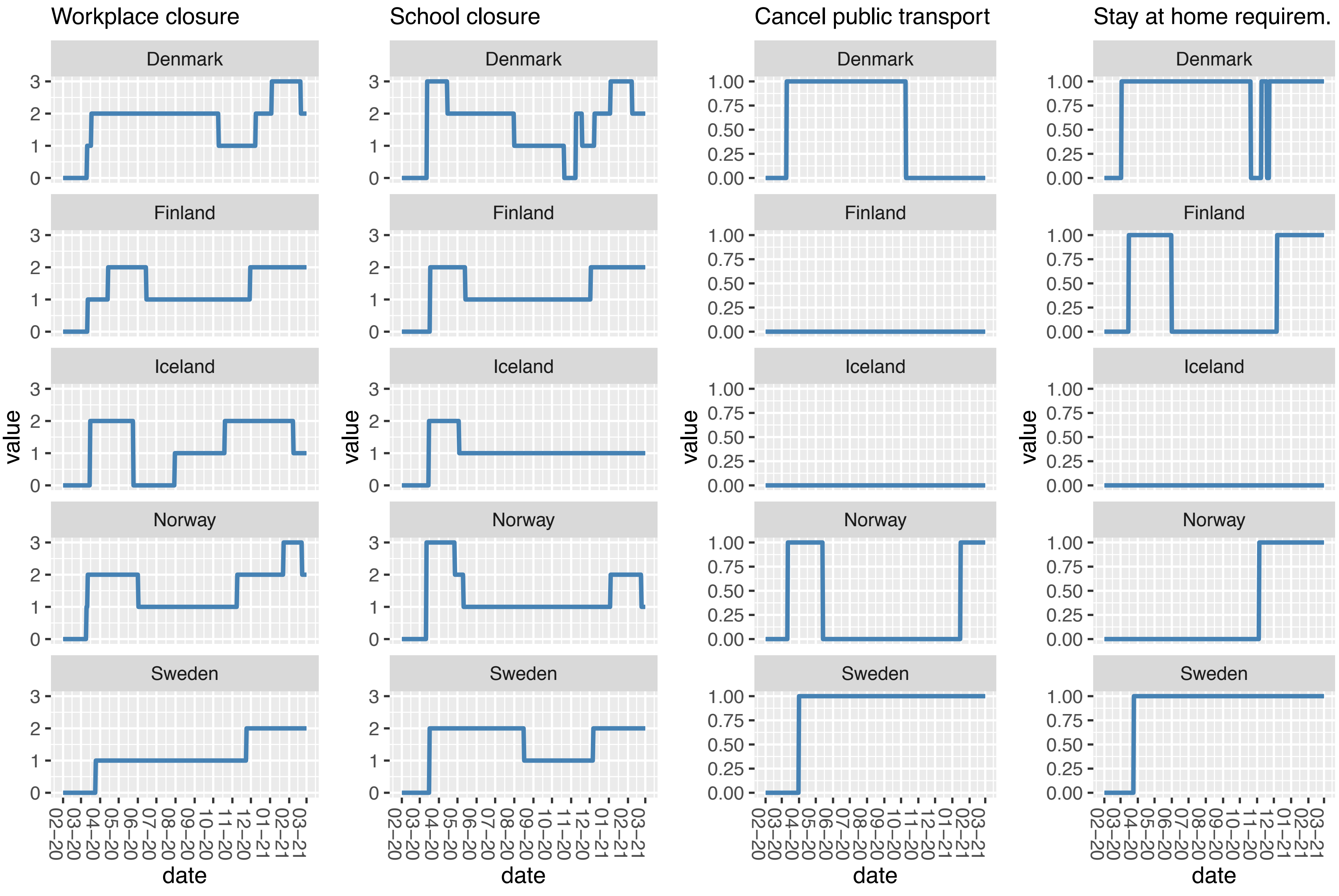

Supplementary Figure S4. Information on indicators of government response between February 2020 through March 2021 by country regarding (i) facemasks, (ii) gathering restrictions, (iii) cancelation of public events, and (iv) restrictions on movement.

This supplementary material is hosted by Eurosurveillance as supporting information alongside the article "the impact of early public health interventions during the first year of SARS-CoV-2 transmission and evolution in northern Europe", on behalf of the authors, who remain responsible for the accuracy and appropriateness of the content. The same standards for ethics, copyright, attributions and permissions as for the article apply. Supplements are not edited by Eurosurveillance and the journal is not responsible for the maintenance of any links or email addresses provided therein.

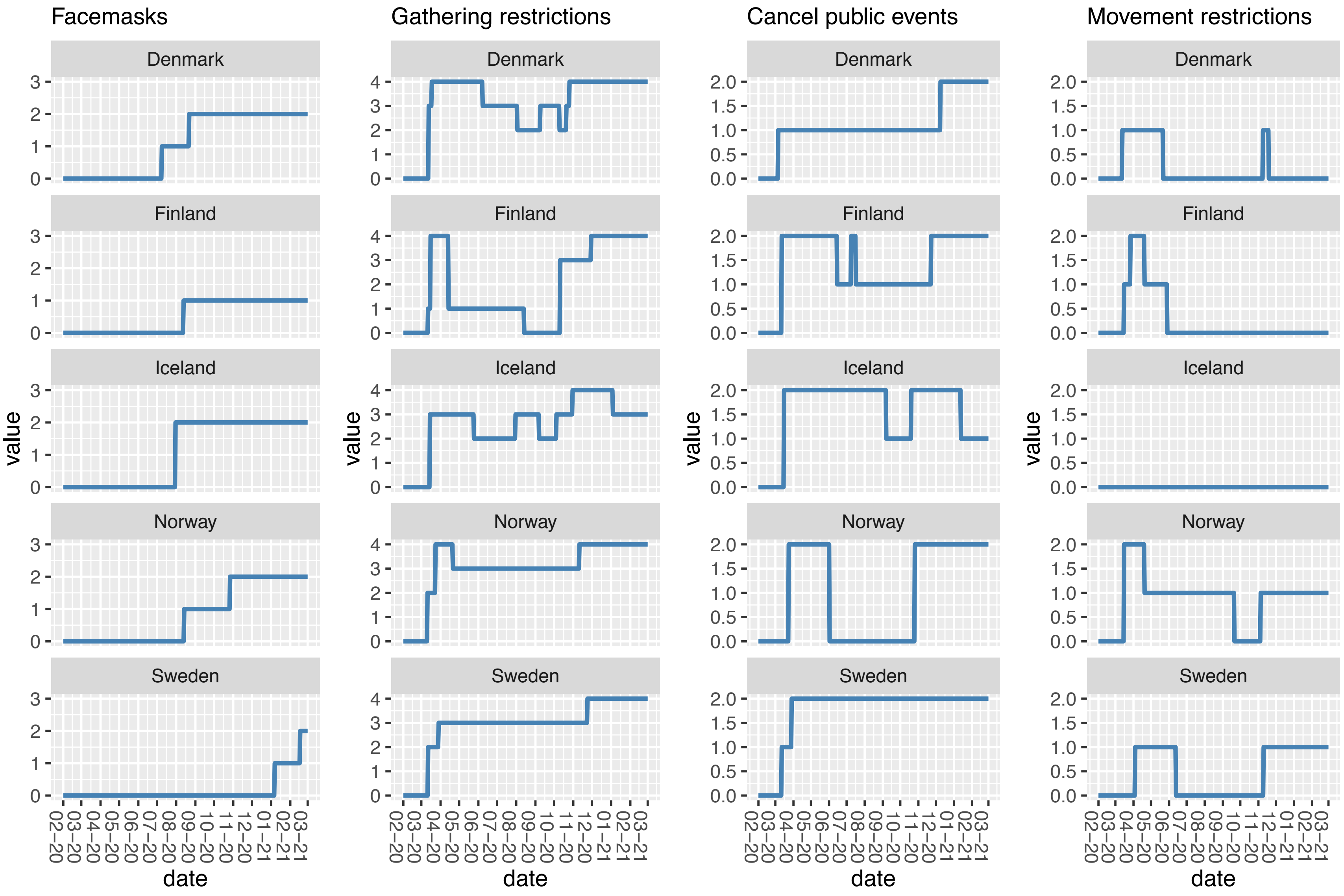

Supplementary Figure S5. Stringency index of government response between February 2020 through March 2021 by country.

This supplementary material is hosted by Eurosurveillance as supporting information alongside the article "the impact of early public health interventions during the first year of SARS-CoV-2 transmission and evolution in northern Europe", on behalf of the authors, who remain responsible for the accuracy and appropriateness of the content. The same standards for ethics, copyright, attributions and permissions as for the article apply. Supplements are not edited by Eurosurveillance and the journal is not responsible for the maintenance of any links or email addresses provided therein.

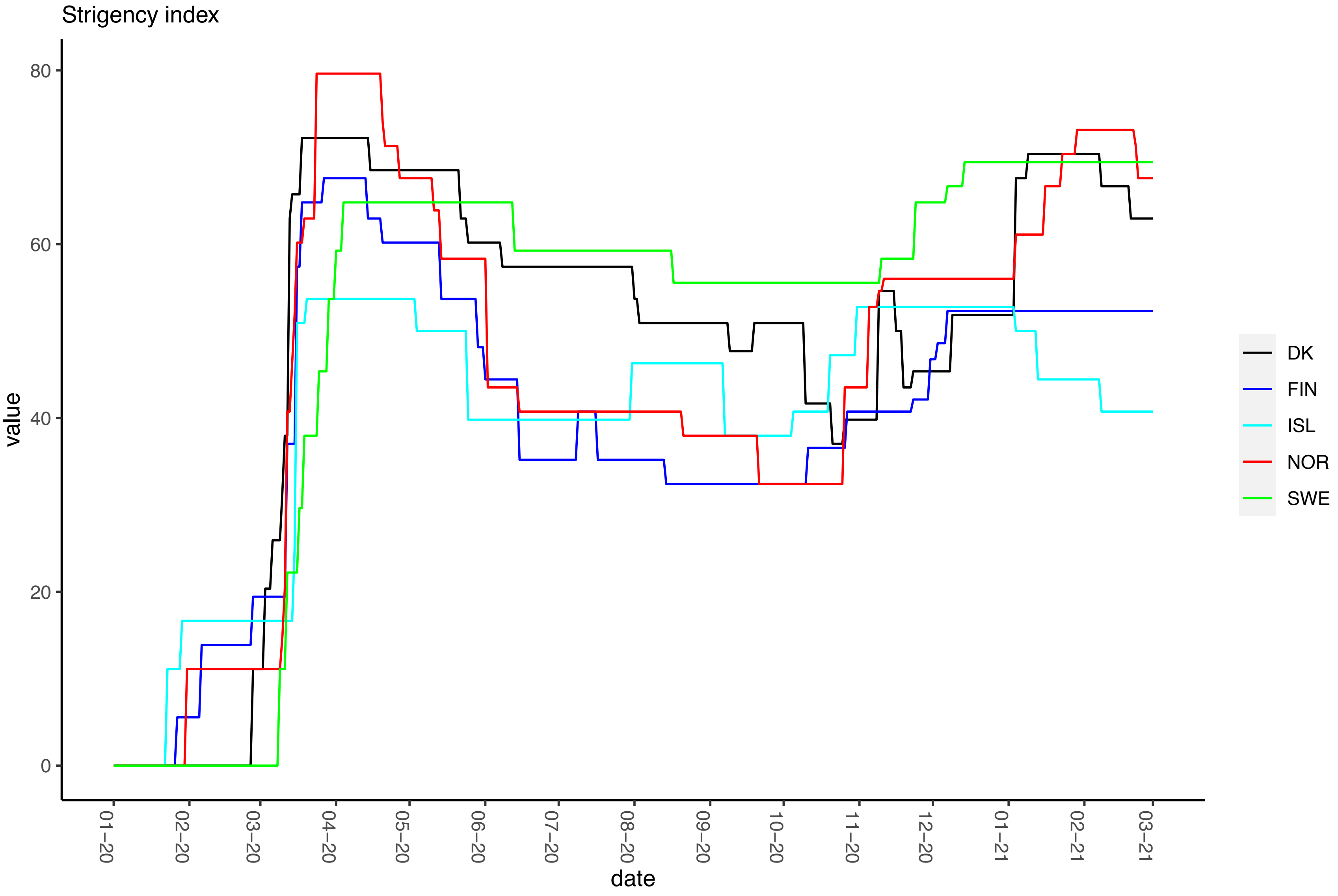

Supplementary Figure S6. Log number of genomes in each of the five most common pangolin lineages and B1.1.7 over time in the complete dataset.

This supplementary material is hosted by Eurosurveillance as supporting information alongside the article "the impact of early public health interventions during the first year of SARS-CoV-2 transmission and evolution in northern Europe", on behalf of the authors, who remain responsible for the accuracy and appropriateness of the content. The same standards for ethics, copyright, attributions and permissions as for the article apply. Supplements are not edited by Eurosurveillance and the journal is not responsible for the maintenance of any links or email addresses provided therein.

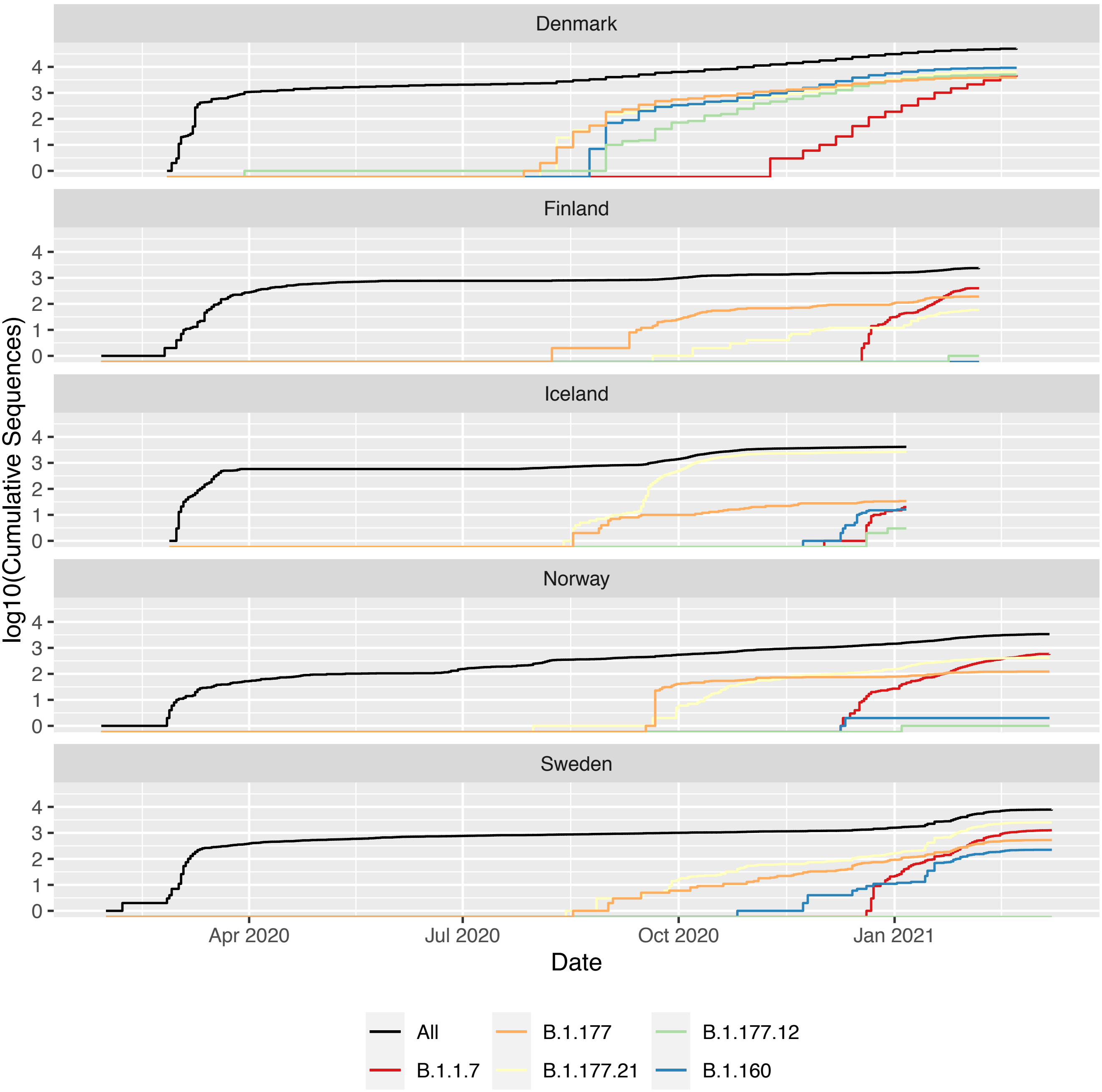

Supplementary Figure S7. Number of putative introductions identified by detecting transmission chains (monophyletic groups of at least 2 genomes from a Nordic country) or singletons (genomes from a Nordic country sitting outside a transmission chains).

This supplementary material is hosted by Eurosurveillance as supporting information alongside the article "the impact of early public health interventions during the first year of SARS-CoV-2 transmission and evolution in northern Europe", on behalf of the authors, who remain responsible for the accuracy and appropriateness of the content. The same standards for ethics, copyright, attributions and permissions as for the article apply. Supplements are not edited by Eurosurveillance and the journal is not responsible for the maintenance of any links or email addresses provided therein.

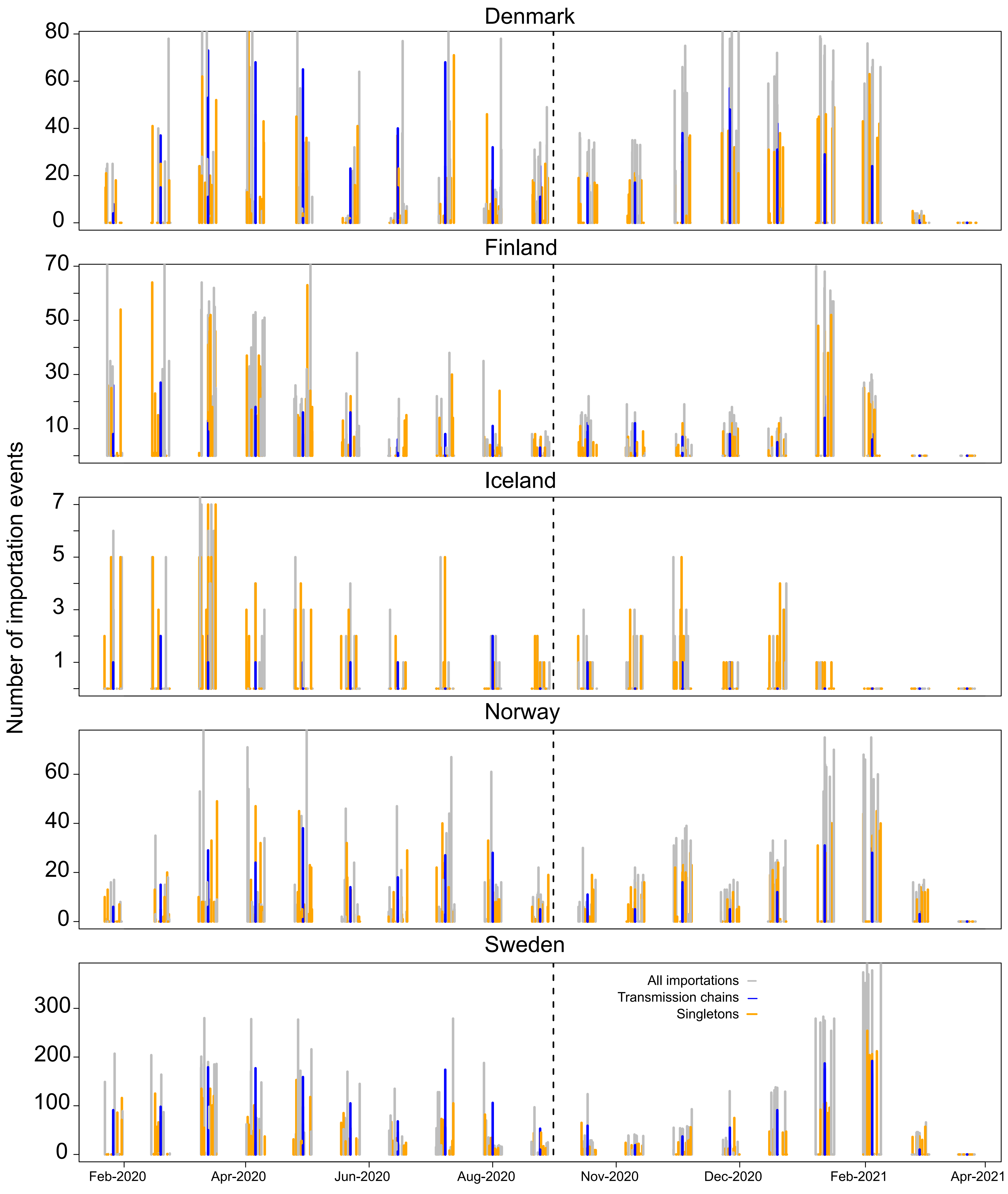

Supplementary Figure S8. Jittered markov rewards for each Nordic country and for the global diversity in the data (labelled as "other").

This supplementary material is hosted by Eurosurveillance as supporting information alongside the article "the impact of early public health interventions during the first year of SARS-CoV-2 transmission and evolution in northern Europe", on behalf of the authors, who remain responsible for the accuracy and appropriateness of the content. The same standards for ethics, copyright, attributions and permissions as for the article apply. Supplements are not edited by Eurosurveillance and the journal is not responsible for the maintenance of any links or email addresses provided therein.

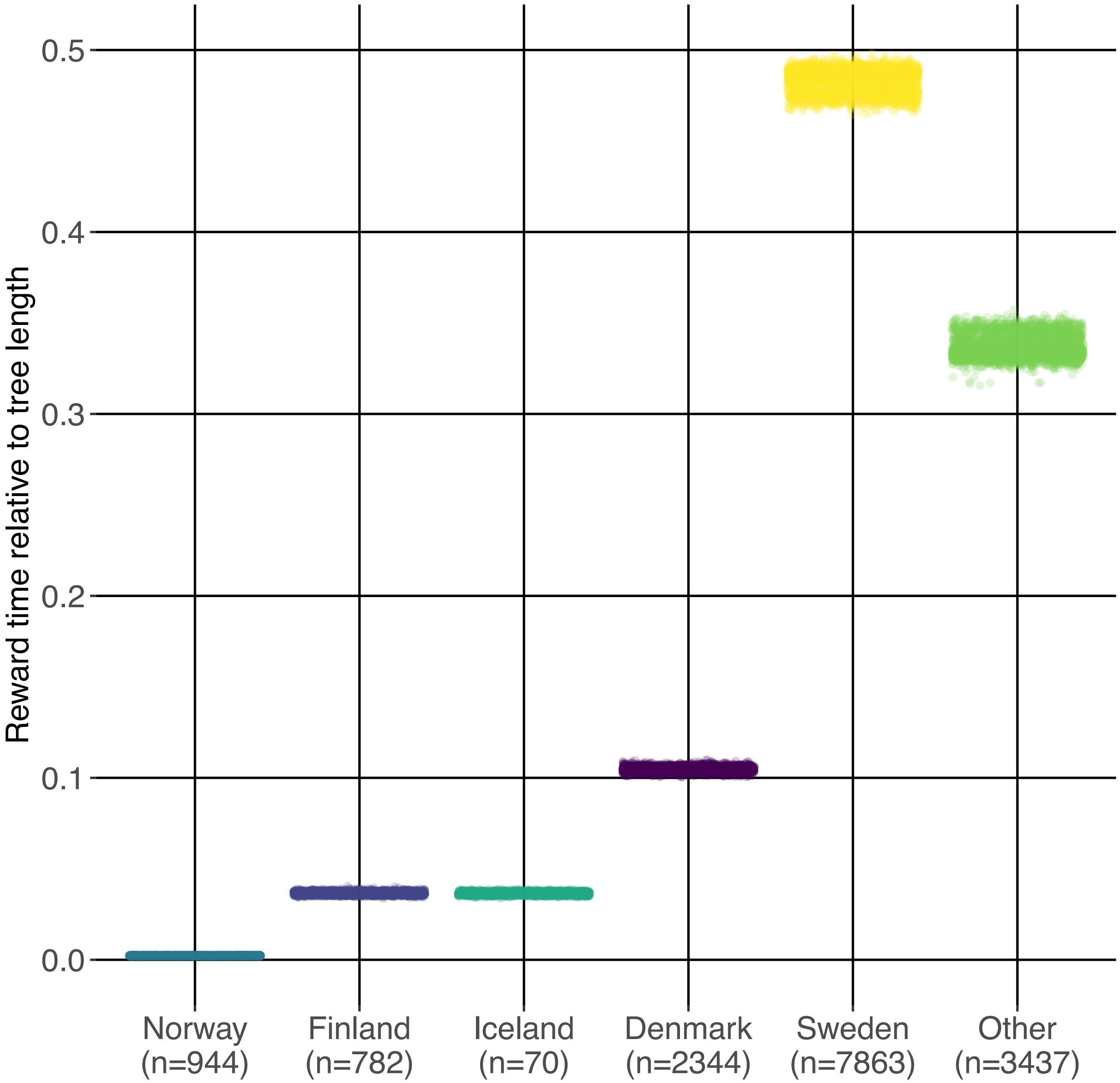

Supplement: Supplementary_figures [file 20-01996_PETTERSSON_Supplementary_figures_S1-S8.pdf]
